# Exploring educational supervisors’ professional commitment: The roles of psychological capital, psychological empowerment, and professional competence

**DOI:** 10.1371/journal.pone.0357360

**Published:** 2026-08-31

**Authors:** Abdulaziz Mohammed Alismail, Abdulaziz Rashed Alghamdi

**Affiliations:** 1 Department of Education and Psychology, College of Education, King Faisal University, Al-Ahsa, Saudi Arabia; 2 Ministry of Education, Saudi Arabia; Zhejiang University, CHINA

## Abstract

**Background:**

Educational supervisors play a pivotal role in teacher development and school improvement, yet little research has examined the psychological mechanisms associated with their professional commitment, particularly in non-Western contexts. Grounded in Conservation of Resources (COR) theory and Self-Determination Theory (SDT), this study examines how psychological capital (PsyCap) relates to professional commitment directly and indirectly through psychological empowerment (a motivational pathway) and professional competence (a capability pathway) among educational supervisors in Saudi Arabia.

**Method:**

A cross-sectional survey was administered to a convenience sample of 358 educational supervisors across the 10 educational districts of the Eastern Province of Saudi Arabia. Structural equation modelling was conducted using robust maximum likelihood estimation (MLR) with Satorra–Bentler corrections in R (lavaan 0.6–17), and indirect effects were tested with bias-corrected bootstrap confidence intervals (5,000 resamples).

**Results:**

The measurement and structural models showed good fit (structural model: Satorra–Bentler χ²(60) = 65.87, p = .281, CFI = .998, TLI = .997, RMSEA = .017, 90% CI [.000,  .037]). Psychological capital was positively associated with psychological empowerment (β = .68, p < .001) and professional competence (β = .72, p < .001), but empowerment was not associated with competence (β = −.06, p = .366). Psychological capital, empowerment, and competence were each directly associated with professional commitment (β = .23,  .39, and  .34, respectively; all p ≤ .001). Psychological capital also showed significant parallel indirect associations with commitment through empowerment (β = .26, 95% CI [.19,  .34]) and through competence (β = .25, 95% CI [.15,  .34]); the serial pathway through both mediators in sequence was not significant (β = −.02, 95% CI [−.05,  .02]). The model accounted for 46.4% of variance in empowerment, 46.2% in competence, and 65.8% in professional commitment.

**Conclusion:**

These findings are consistent with a dual-pathway model in which psychological resources are associated with professional commitment through largely independent motivational and capability-based mechanisms. Because the design is cross-sectional and based on self-report, these associations should not be interpreted as causal; they may instead help justify testing psychological-capital-focused interventions, empowerment-supportive leadership practices, and professional-development investments in future longitudinal or intervention research, including within reform contexts such as Saudi Arabia’s Vision 2030. Limitations and directions for future research are discussed.

## Introduction

Professional commitment refers to an individual’s enduring identification with and dedication to their profession, extending beyond organisational attachment to include adherence to professional values, occupational identity, and long-term career involvement [1,2]. In educational settings, professional commitment predicts job satisfaction, instructional quality, resilience, reduced turnover intentions, and stronger organisational effectiveness [1,3]. Professional commitment among educational supervisors constitutes a critical determinant of educational system effectiveness. Positioned at the intersection of policy implementation and instructional practice, supervisors shape teacher development, ensure curriculum fidelity, and support school improvement initiatives. Within the Saudi Arabian education system, supervisors play a pivotal role in translating national reforms into school-level practices, mentoring teachers, and monitoring instructional quality in alignment with national standards. Their sustained professional dedication influences instructional quality, organisational coherence, and ultimately student outcomes [4,5]. Among educational supervisors, maintaining professional commitment may be particularly challenging because of expanding administrative duties, policy implementation pressures, and the need to balance evaluative and developmental responsibilities. These demands may undermine sustained motivation unless supervisors possess sufficient psychological and professional resources. In periods of systemic reform, technological transformation, and increasing accountability demands, maintaining supervisors’ professional commitment becomes particularly essential.

Educational systems globally are undergoing rapid restructuring driven by digital innovation, policy reform, and evolving societal expectations. In Saudi Arabia, these transformations are concretely embodied in Vision 2030, which emphasises human capital development, digital integration, and educational quality enhancement [5]. For educational supervisors, these reforms introduce expanded responsibilities such as overseeing digital learning platforms, supporting competency-based curricula, and ensuring alignment with national performance indicators. While these changes create opportunities for professional growth and strategic influence, they also generate challenges including role overload, continuous skill adaptation, and increased accountability pressures. These conditions make the sustainability of professional commitment both more complex and more critical.

Nevertheless, engagement and commitment gaps among supervisory staff may translate into reduced supervisory effectiveness, limited instructional innovation, and weaker reform implementation. Despite this, limited empirical research has examined the psychological mechanisms that sustain professional commitment among educational supervisors, particularly within reform-intensive and non-Western contexts such as Saudi Arabia [6].

### Theoretical foundation and conceptual logic

This study adopts an integrated theoretical perspective combining **Self-Determination Theory (SDT)** and **Conservation of Resources (COR) theory**, each contributing distinct but complementary explanatory power. SDT explains the **motivational processes** underlying sustained professional engagement, while COR theory explains how **personal psychological resources** enable individuals to cope with demands and maintain functioning under pressure.

Self-Determination Theory (SDT) posits that sustained motivation emerges when individuals experience satisfaction of three basic psychological needs: autonomy, competence, and relatedness [7]. This framework is particularly relevant to educational supervisors, whose work requires independent judgement, advanced professional expertise, and continuous collaboration with teachers, school leaders, and policymakers. When these needs are satisfied, supervisors may be more likely to internalise professional values and maintain long-term occupational commitment. However, in high-demand reform environments, motivation alone may not be sufficient; supervisors also require psychological resources and professional capability to cope with role pressures effectively.

COR theory complements this perspective by emphasising that individuals rely on psychological resources to manage stress and sustain performance [8]. In reform-intensive contexts such as Saudi Arabia, supervisors must not only be motivated but also possess sufficient psychological and professional resources to cope with expanding demands. In this study, psychological capital (PsyCap) is conceptualised as a foundational personal resource, while psychological empowerment and professional competence represent mechanisms through which these resources are translated into sustained professional commitment. Together, these frameworks provide a coherent explanation: PsyCap (resource base) enables individuals to cope with demands (COR), empowerment (motivational state) reflects fulfilment of psychological needs (SDT), competence (capability) enables effective role performance. These processes collectively sustain professional commitment.

### Psychological capital and its downstream effects

Psychological capital (PsyCap) comprises hope, self-efficacy, resilience, and optimism [9]. In the context of educational supervision, these components manifest in concrete ways: hope supports goal-directed reform implementation, self-efficacy enhances confidence in mentoring teachers, resilience enables adaptation to policy changes, and optimism sustains positive expectations about reform outcomes.

From a COR perspective, PsyCap represents a key personal resource that individuals draw upon to manage demands and avoid resource depletion. Individuals with higher PsyCap are more likely to invest effort, persist under pressure, and maintain engagement. Empirical evidence associates PsyCap with well-being, lower burnout, and sustained professional functioning [10,11]. In addition, PsyCap is associated with adaptive organisational behaviours and positive work attitudes that are relevant in reform-driven environments [12,13].

Importantly, PsyCap is not only a coping resource but also a resource-generating mechanism, enabling the development of further motivational and capability-based states. First, individuals with high PsyCap are more likely to experience a sense of control, meaning, and confidence in their roles, which aligns closely with psychological empowerment. Based on this theoretical and empirical evidence, the following hypothesis is proposed:

**H1: Psychological capital is positively associated with psychological empowerment.**

PsyCap may be associated with proactive learning, skill acquisition, and adaptive behaviour, which correspond with professional competence. Supervisors with high self-efficacy and resilience, for example, may be more likely to engage in continuous professional development and apply new knowledge in practice. Accordingly, the following hypothesis is advanced:

**H2: Psychological capital is positively associated with professional competence.**

### Psychological empowerment as a motivational mechanism

Psychological empowerment refers to a motivational state characterised by meaning, competence perception, self-determination, and impact [14]. In educational supervision, empowerment is reflected in supervisors’ perceptions that their work is meaningful, that they have discretion in decision-making, and that their actions influence educational outcomes.

From an SDT perspective, psychological empowerment reflects fulfilment of autonomy and competence needs and is therefore expected to correspond with stronger intrinsic motivation [7]. Empirical evidence indicates that empowered professionals report stronger engagement, job satisfaction, organisational commitment, innovation, and proactive behaviour [12,15–18]. Empowerment may also be associated with professional competence development because supervisors who feel autonomous and impactful may be more likely to take initiative, engage in problem-solving, and pursue skill refinement. Consistent with these arguments, the following hypothesis is proposed:

**H3: Psychological empowerment is positively associated with professional competence.**

In addition, empowered supervisors may be more likely to internalise professional goals and remain psychologically invested in their occupation, thereby reporting stronger commitment [19]. Thus, the following hypothesis is formulated:

**H4: Psychological empowerment is positively associated with professional commitment.**

### Professional competence as a capability-based mechanism

Professional competence encompasses technical expertise, pedagogical knowledge, decision-making ability, and interpersonal skills such as communication and conflict resolution [19]. In Saudi Arabia, competence development is increasingly emphasised through structured training initiatives, continuous professional development programmes, and digital skill enhancement aligned with Vision 2030.

Professional competence functions as a capability-based construct linking psychological resources with sustained professional outcomes. From a COR perspective, competence represents a valuable personal resource associated with individuals’ capacity to meet job demands [8]. From an SDT perspective, competence satisfaction is expected to correspond with intrinsic motivation and professional identity.

Empirical evidence suggests that competence predicts organisational effectiveness, strengthens the relationship between self-efficacy and commitment, and mediates relationships between psychological resources and professional outcomes [8,13,14,19–22].

Therefore:

**H5: Professional competence is positively associated with professional commitment**

#### Direct association between psychological capital and professional commitment.

Beyond its possible indirect associations through empowerment and competence, psychological capital may also be directly associated with professional commitment. Individuals with strong hope, self-efficacy, resilience, and optimism may report sustained occupational dedication independent of their current levels of empowerment or perceived competence, consistent with evidence that psychological capital is broadly associated with positive work attitudes and outcomes [12,15,18]. Accordingly, the following hypothesis is proposed:

**H6: Psychological capital is positively and directly associated with professional commitment.**

### Indirect effects and integrated mechanism

In addition to these direct relations, psychological capital is expected to be indirectly associated with professional commitment through psychological empowerment (a motivational pathway) and professional competence (a capability pathway). Because empowerment was also hypothesised to be associated with competence (H3), three conceptually distinct indirect pathways can be distinguished: two parallel pathways, in which psychological capital is associated with commitment through empowerment alone or competence alone, and one sequential pathway, in which psychological capital is associated with empowerment, which in turn is associated with competence and then commitment. Separating these pathways clarifies whether empowerment and competence function as independent or sequential statistical mechanisms linking psychological resources with commitment [22]. Accordingly, the following indirect hypotheses are proposed:

**H7a: Psychological capital has a positive indirect association with professional commitment through psychological empowerment.**

**H7b: Psychological capital has a positive indirect association with professional commitment through professional competence.**

**H7c: Psychological capital has a positive sequential indirect association with professional commitment through psychological empowerment and then professional competence.**

### Research gap and contribution

Although prior research has established the individual importance of PsyCap, psychological empowerment, and professional competence, these constructs have rarely been integrated into a single explanatory framework. Existing studies tend to focus on isolated relationships, limiting understanding of how psychological resources are linked with sustained professional commitment.

This gap is particularly evident in the context of educational supervision, which remains underexplored despite its strategic importance in reform implementation. Furthermore, research in non-Western contexts—especially Saudi Arabia—is limited, despite the unique institutional, cultural, and policy dynamics shaping professional roles under Vision 2030 [23,24]. By integrating PsyCap, psychological empowerment, and professional competence into a unified structural model, this study provides a theoretically grounded and contextually relevant explanation of professional commitment among educational supervisors.

### Proposed model

**Fig 1:**
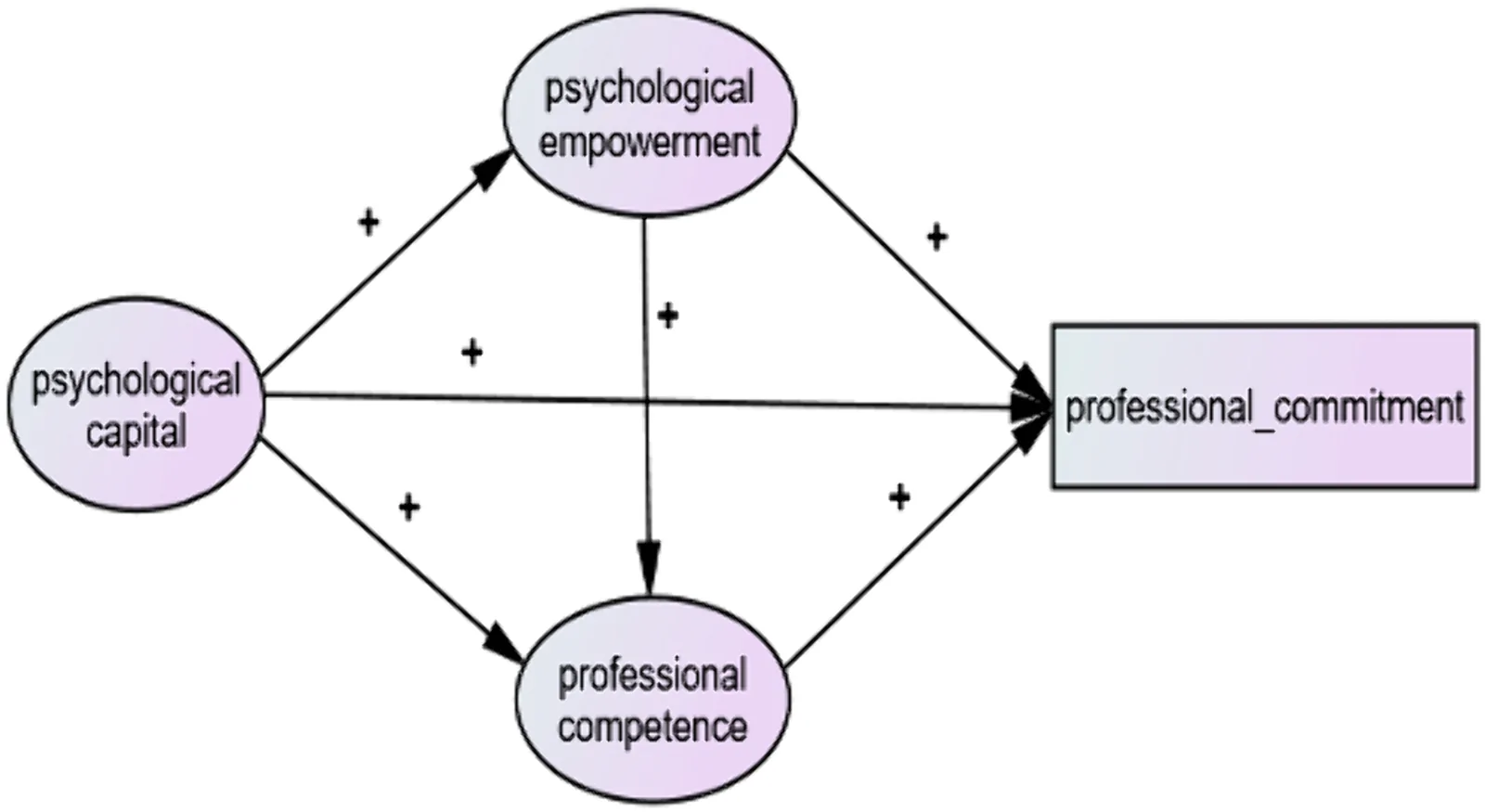
Proposed model of the determinants of educational supervisors’ professional commitment. Note: The proposed model depicts hypothesised positive associations (H1–H6) among psychological capital, psychological empowerment, professional competence, and professional commitment, including the direct paths from psychological capital and from psychological empowerment to professional commitment.

Based on the theoretical and empirical foundations discussed above, the proposed structural model is presented in Fig 1.

### Study Purpose

The present study examines the structural relationships among psychological capital, psychological empowerment, professional competence, and professional commitment among educational supervisors in Saudi Arabia using structural equation modelling. It specifically tests whether psychological capital is associated with professional commitment directly and indirectly through motivational and capability-based pathways.

## Method

### Participants and sampling procedure

#### Population.

The target population for this study comprised all educational supervisors employed by the Ministry of Education in the Eastern Province of Saudi Arabia. Educational supervisors are professional educators responsible for monitoring and supporting teacher performance, ensuring curriculum implementation, evaluating instructional quality, and facilitating professional development across schools within assigned districts. The Eastern Province, one of Saudi Arabia’s 13 administrative regions, includes 10 educational districts serving urban, suburban, and semi-urban communities. The region was selected due to its demographic diversity, representation of varied educational contexts, and logistical accessibility for data collection. At the time of the study, approximately 7,100 educational supervisors were employed across the region, forming the accessible population.

### Sample and sampling technique

A convenience sampling technique was employed to recruit participants from the accessible population. This approach was adopted due to logistical constraints, including the absence of a centralized sampling frame, geographical dispersion of supervisors across districts, and time limitations during data collection. While convenience sampling enables efficient access to participants in large and distributed populations, it introduces limitations related to representativeness and generalisability. Specifically, the findings of this study should be interpreted with caution, as they may reflect the characteristics of those who were more accessible or willing to participate rather than the entire population of educational supervisors in Saudi Arabia. Potential self-selection bias and overrepresentation of more engaged or digitally accessible supervisors are acknowledged as methodological limitations. However, the inclusion of supervisors from all 10 educational districts helped to partially reduce sampling bias by ensuring heterogeneity across institutional and geographic contexts. Convenience sampling remains widely used in educational and organisational research where full probability sampling is impractical [25].

### Participant recruitment and data collection

Participants were recruited through coordination with educational district offices and professional communication channels facilitated by the Eastern Province Education Directorate. Invitation letters detailing the study purpose, voluntary participation, confidentiality, and estimated completion time (25–30 minutes) were distributed via official administrative platforms and professional networks. The recruitment period for this study started on 25/05/2025 and ended on 30/07/2025. A total of 392 responses were received. After screening for completeness and eligibility, 34 responses were excluded due to missing data or ineligibility (e.g., trainees or inactive supervisors). The final sample consisted of 358 valid responses with complete data.

The sample size exceeds recommended thresholds for structural equation modelling (SEM), which typically suggest 10–20 observations per estimated parameter or a minimum of 200 cases for complex models [26,27].

### Participant characteristics and analytical relevance

The final sample (N = 358) consisted of 61.7% male and 38.3% female supervisors. This distribution reflects the gendered structure of the Saudi educational system, where historical staffing patterns and institutional segregation have influenced supervisory representation. Gender distribution is analytically relevant because supervisory experiences, leadership expectations, and interactional roles may vary across gender groups within the educational context. Regarding professional experience, participants ranged from 1 to over 21 years of service. Professional experience is particularly relevant in this study because it is likely to influence psychological capital, empowerment perceptions, competence development, and commitment levels. More experienced supervisors may exhibit higher role mastery and resilience, while less experienced supervisors may rely more on psychological resources such as empowerment and optimism. Participants were drawn from all 10 educational districts in the Eastern Province, ensuring geographic diversity across institutional contexts (Table 1).

**Table 1:** Demographic and professional characteristics of study participants (N = 358).

Characteristic	Category	Frequency (n)	Percentage (%)
Gender	Male	221	61.7
Gender	Female	137	38.3
Years of experience	1–5 years	53	14.8
Years of experience	6–10 years	115	32.1
Years of experience	11–15 years	94	26.3
Years of experience	16–20 years	60	16.8
Years of experience	More than 21 years	36	10.1
Educational district	Al-Khafji	31	8.7
Educational district	An-Nu'ayriyah	32	8.9
Educational district	Buqayq	36	10.1
Educational district	Jubail	20	5.6
Educational district	Khobar	35	9.8
Educational district	Ras Tanura	33	9.2
Educational district	Sharq	40	11.2
Educational district	Gharb	66	18.4
Educational district	Qaryat Al Ulya	28	7.8
Educational district	Qatif	37	10.3

Note: Percentages may not sum to 100% due to rounding.

### Ethical considerations

Ethical approval was obtained from the Research Ethics Committee of King Faisal University (Reference: KFU-REC-2025-MAY-ETHICS3455). Participation was voluntary, and informed consent was obtained from all participants prior to data collection. The confidentiality and anonymity of participants were ensured, and all data were securely stored in accordance with ethical research standards and the Declaration of Helsinki.

### Instruments

All instruments were administered in Arabic using a five-point Likert scale (1 = never to 5 = always).

### Translation and cultural adaptation

All instruments originally developed in English were translated into Arabic using a forward–backward translation procedure. Two bilingual experts independently translated the instruments into Arabic, and a third bilingual expert performed back-translation into English. Discrepancies were resolved through consensus to ensure semantic equivalence. In addition to linguistic equivalence, cultural adaptation was carefully considered. Items were reviewed to ensure cultural relevance within the Saudi educational context, particularly regarding supervisory authority structures, workplace interaction norms, and hierarchical decision-making practices. For example, wording related to autonomy and decision-making was adjusted to reflect the collective and administratively structured nature of school supervision in Saudi Arabia. A pilot test involving 20 educational supervisors confirmed clarity, cultural appropriateness, and item comprehensibility. Minor modifications were made to improve contextual relevance without altering construct meaning.

### Psychological capital scale (PsyCap)

Psychological capital was measured using the 24-item Psychological Capital Questionnaire (PCQ) developed by Luthans et al. [9]. The scale assesses four dimensions: self-efficacy, hope, resilience, and optimism (6 items per dimension). Reliability coefficients in the present study were satisfactory:

- Self-efficacy (α = .815)
- Hope (α = .789)
- Resilience (α = .859)
- Optimism (α = .833)
- Overall PsyCap (α = .882)

Confirmatory factor analysis supported the four-factor structure. (Note: The instrument consists of 24 items, consistent with the standard validated PCQ version.)

### Psychological empowerment Scale (PE)

Psychological empowerment was measured using Spreitzer’s 12-item Psychological Empowerment Scale [14], covering meaning, competence, self-determination, and impact. Reliability indices ranged from α = .704 to  .901 across subscales, with overall reliability of α = .896. CFA confirmed acceptable model fit.

### Professional competence scale (PCo)

Professional competence was assessed using a 25-item scale [28], measuring work achievement, performance development, social interaction, and coping with stress. Internal consistency ranged from α = .778 to  .882, with total reliability of α = .890. CFA confirmed acceptable construct validity.

### Professional commitment scale (PC)

Professional commitment was measured using a 5-item scale [29] assessing occupational identification and dedication. Reliability was acceptable (α = .774). Because item-level responses were not separable into distinct sub-dimensions, professional commitment was represented in the structural model as a single observed composite (summed) score rather than as a multi-indicator latent factor; this modelling choice is described further in the Statistical Analysis section.

### Statistical analysis

#### Preliminary analysis.

Data were analysed using SPSS 26 and R. No missing data were present in the final dataset. Normality tests indicated deviations from multivariate normality; therefore, robust estimation methods were applied.

### Structural equation modelling

Structural equation modelling (SEM) was conducted using robust maximum likelihood estimation with Satorra–Bentler corrections to account for non-normality [30]. Likert-scale responses were treated as continuous variables, consistent with methodological recommendations for five-point scales under robust estimation [31].

### Measurement model evaluation

Convergent validity was assessed using Composite Reliability (CR ≥ 0.70) and Average Variance Extracted (AVE ≥ 0.50). Constructs with AVE slightly below thresholds were retained when CR exceeded acceptable limits [32]. Discriminant validity was assessed using the Fornell–Larcker criterion.

### Model fit evaluation

Model fit was evaluated using multiple indices: Satorra–Bentler χ², CFI (≥ 0.92), and RMSEA (≤ 0.07). Given χ² sensitivity to sample size, greater emphasis was placed on CFI and RMSEA [33].

### Common method bias

Procedural remedies included anonymity assurance, item separation, and neutral instructions. An unrotated principal-axis factor analysis indicated that the first factor accounted for 45.2% of the total variance, below the 50% threshold [34]. A single-factor CFA also demonstrated poor fit relative to the hypothesised measurement model, indicating that common method bias was unlikely to be a serious concern.

## Results

### Measurement model

The measurement model was assessed using confirmatory factor analysis (CFA) to evaluate the adequacy of three latent constructs: Psychological Capital (PsyCap), Psychological Empowerment (PE), and Professional Competence (PCo). Professional Commitment (PC) was represented as a single observed composite score rather than as a latent construct (see Measures) and was therefore not included in this CFA. All standardized factor loadings were statistically significant (p < .001) and exceeded the recommended threshold of 0.50 [33], ranging from 0.707 to 0.848. This indicates that all observed indicators adequately represent their respective latent constructs, demonstrating strong indicator reliability and convergent validity. No items were removed, suggesting that the measurement instruments were culturally and contextually appropriate for the Saudi educational supervision context. Overall, the CFA results confirm that the three latent constructs (psychological capital, psychological empowerment, and professional competence) are empirically distinct yet well-measured by their indicators (Table 2). Professional commitment, represented as a single observed composite score, is not part of this measurement model (see Measures).

**Table 2:** Standardized factor loadings for the measurement model (n = 358).

Latent Variable	Indicator	Standardized loading	SE	p
Psychological Capital	self_efficacy	0.800	0.024	< .001
Psychological Capital	hope	0.707	0.031	< .001
Psychological Capital	resilience	0.825	0.022	< .001
Psychological Capital	optimism	0.733	0.028	< .001
Psychological Empowerment	meaning	0.819	0.023	< .001
Psychological Empowerment	competence perception	0.770	0.026	< .001
Psychological Empowerment	self_determination	0.740	0.028	< .001
Psychological Empowerment	impact	0.821	0.022	< .001
Professional Competence	work_achievement	0.848	0.022	< .001
Professional Competence	performance_development	0.751	0.030	< .001
Professional Competence	social_interaction	0.724	0.029	< .001
Professional Competence	stress_management	0.823	0.021	< .001

Note. n = 358. Loadings are from the three-factor confirmatory measurement model (Satorra–Bentler χ²(51) = 60.93, p = .161; CFI = .996; RMSEA = .023). Professional commitment was represented as a single observed composite score (Cronbach’s α = .774) rather than as a latent factor and is therefore not included in this table; see Measures and Table 3.

### Measurement model fit

The measurement model, comprising three latent constructs (psychological capital, psychological empowerment, and professional competence, each with four indicators), demonstrated excellent fit to the data: Satorra–Bentler χ²(51) = 60.93, p = .161; CFI = .996; TLI = .994; RMSEA = .023 (90% CI [.000,  .043]); SRMR = .029. Professional commitment was modelled as a single observed composite score rather than as a multi-indicator latent factor (see Measures), and was therefore not included in this measurement model; its correlations with the three latent constructs are reported in Table 3. These indices indicate that the hypothesised three-factor structure provides a very good representation of the observed data, supporting the validity of the measurement model. A single-factor model including all indicators plus the professional commitment composite was also tested to assess common method bias. This model fit the data poorly (CFI = .753; RMSEA = .166) relative to the hypothesised model, and an unrotated principal-axis factor analysis showed that a single factor accounted for 45.2% of the total variance, below the 50% threshold; together, these results indicate that common method variance is unlikely to account for the observed relationships [34].

**Table 3:** Composite reliability, average variance extracted, and discriminant validity (n = 358).

Construct	CR	AVE	1. PsyCap	2. PE	3. PCo	4. PC
1. Psychological Capital (PsyCap)	0.851	0.589	(0.768)			
2. Psychological Empowerment (PE)	0.868	0.622	0.681**	(0.788)		
3. Professional Competence (PCo)	0.867	0.621	0.677**	0.428**	(0.788)	
4. Professional Commitment (PC)	—	—	0.724**	0.689**	0.661**	—

Note. n = 358. CR = composite reliability; AVE = average variance extracted. Diagonal values in parentheses are the square roots of AVE for the three latent constructs; off-diagonal values are latent factor correlations. Discriminant validity via the Fornell–Larcker criterion is supported: each square-root-AVE diagonal value exceeds its corresponding off-diagonal correlations. HTMT ratios (a complementary discriminant-validity check) were 0.679 (PsyCap–PE), 0.683 (PsyCap–PCo), and 0.436 (PE–PCo), all below the conservative 0.85 threshold. ᵃProfessional commitment (PC) was modelled as a single observed composite score (α = .774) rather than as a latent factor; CR and AVE are therefore not applicable, and its correlations with the three latent constructs are reported for reference rather than compared against square-root-AVE values. **p < .001.

### Structural model

The structural model also demonstrated excellent fit: Satorra–Bentler χ²(60) = 65.87, p = .281; CFI = .998; TLI = .997; RMSEA = .017 (90% CI [.000,  .037]); SRMR = .028. This indicates that the hypothesised theoretical model—linking psychological capital, empowerment, competence, and professional commitment—adequately represents the observed relationships among educational supervisors in Saudi Arabia.

### Direct effects

All path coefficients reported below are fully standardized. Psychological capital showed a strong positive association with psychological empowerment (β = .68, p < .001), supporting H1. This finding indicates that supervisors with higher levels of hope, self-efficacy, resilience, and optimism are more likely to perceive their work as meaningful, autonomous, and impactful.

Similarly, psychological capital was significantly associated with professional competence (β = .72, p < .001), supporting H2. This suggests that supervisors reporting stronger psychological resources also tended to report higher professional skills and capabilities.

Contrary to expectations, psychological empowerment was not significantly associated with professional competence (β = −.06, p = .366); therefore, H3 was not supported. Feeling empowered was not associated with higher self-reported competence in this sample, suggesting that motivational perceptions and perceived professional capability may operate independently in the absence of structural or institutional learning opportunities.

**Fig 2:**
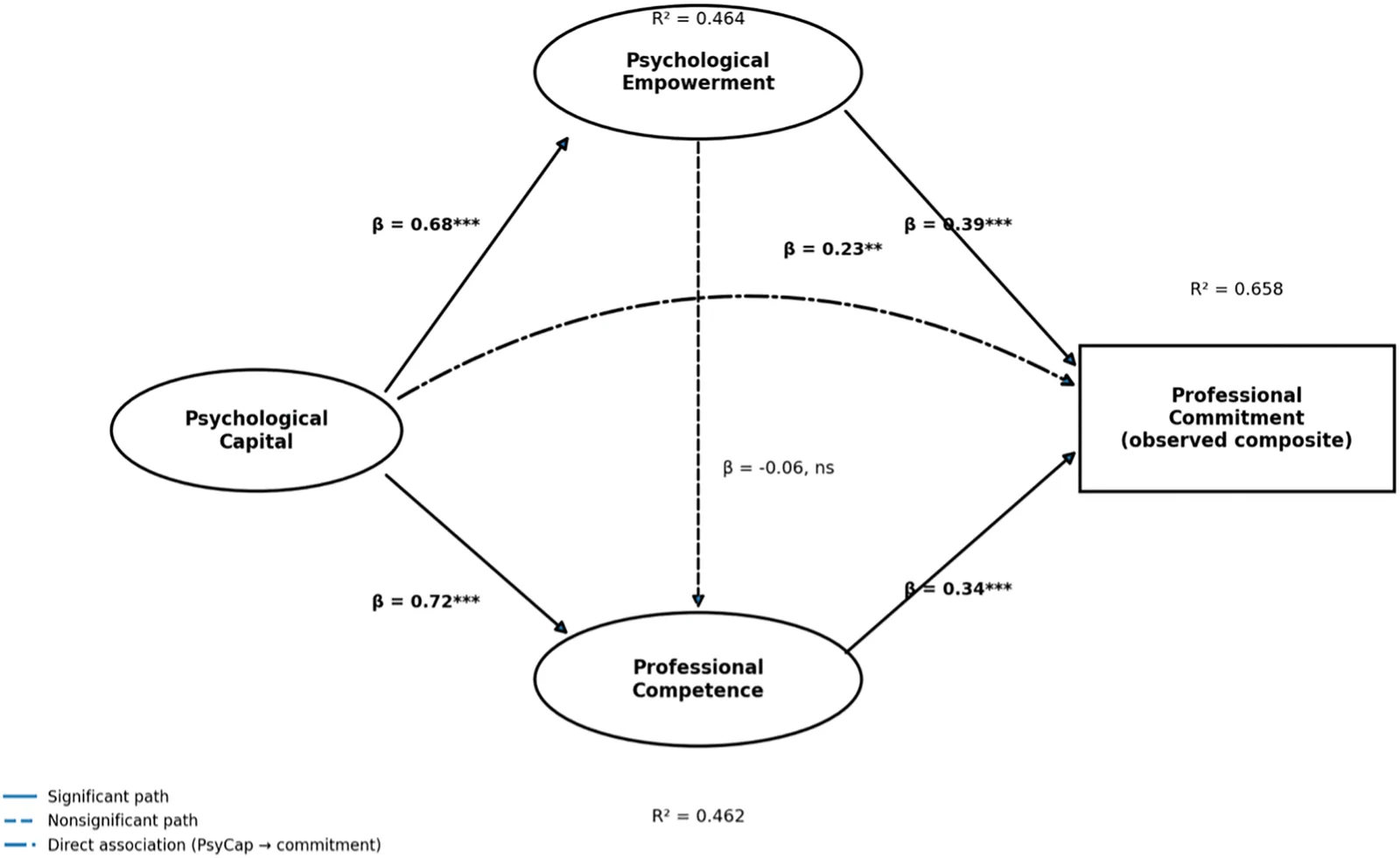
Standardized structural model of psychological capital, psychological empowerment, professional competence, and professional commitment. Coefficients are fully standardized path estimates; the dashed line indicates a nonsignificant path (psychological empowerment → professional competence). *p < .05; **p < .01; ***p < .001. Professional commitment was modelled as a single observed composite score (see Measures).

Psychological empowerment showed a significant direct association with professional commitment (β = .39, p < .001), supporting H4. Professional competence also showed a significant direct association with professional commitment (β = .34, p < .001), supporting H5. In addition, psychological capital showed a significant direct association with professional commitment independent of empowerment and competence (β = .23, p = .001), supporting H6. Together, these results suggest that supervisors who perceive their work as meaningful and autonomous, who report higher professional competence, and who hold stronger psychological resources more generally, each tend to report stronger professional attachment (Fig 2).

### Indirect effects and mediation analysis

Indirect associations were examined using bias-corrected bootstrapping with 5,000 resamples. Unstandardized estimates are reported, with standardized estimates in parentheses. The statistical output supplied with the manuscript did not include the exact bootstrap standard errors and z statistics; these values are marked below for insertion from the original lavaan output before resubmission.

Through psychological empowerment (H7a): B = 0.277, bootstrap SE = [INSERT], z = [INSERT], p < .001, 95% bias-corrected CI [0.195, 0.366] (β = .26, 95% CI [.19,  .34]).

Through professional competence (H7b): B = 0.258, bootstrap SE = [INSERT], z = [INSERT], p < .001, 95% bias-corrected CI [0.167, 0.372] (β = .25, 95% CI [.15,  .34]).

These results support H7a and H7b, indicating that both the motivational (empowerment) and capability-based (competence) pathways carried statistically significant portions of the association between psychological capital and professional commitment and that the two pathways were of comparable magnitude.

The sequential pathway (psychological capital → empowerment → competence → commitment) was not significant: B = −0.015, bootstrap SE = [INSERT], z = [INSERT], p = .392, 95% bias-corrected CI [−0.054, 0.016] (β = −.02, 95% CI [−.05,  .02]). H7c was therefore not supported, consistent with the non-significant empowerment-to-competence path (H3) and with the interpretation that the two mediating pathways operated in parallel rather than in sequence.

The total indirect association of psychological capital with professional commitment was B = 0.519, bootstrap SE = [INSERT], z = [INSERT], p < .001, 95% bias-corrected CI [0.386, 0.663] (β = .49). The total association, comprising the direct and indirect components, was B = 0.762, bootstrap SE = [INSERT], z = [INSERT], p < .001, 95% bias-corrected CI [0.669, 0.863] (β = .72).

### Explained variance

The model accounted for 46.4% of the variance in psychological empowerment, 46.2% of the variance in professional competence, and 65.8% of the variance in professional commitment. These values indicate strong explanatory power, particularly for professional commitment, suggesting that the psychological resources and mechanisms examined here are strongly associated with supervisors’ commitment outcomes.

### Summary of key findings

Overall, the findings are consistent with psychological capital functioning as a central psychological resource associated with professional commitment both directly and indirectly, through psychological empowerment and professional competence. Empowerment and competence were each independently associated with commitment, but did not operate sequentially: the non-significant empowerment-to-competence path suggests that motivational perceptions alone are not associated with higher self-reported competence unless accompanied by structural learning opportunities and professional development systems. Because the design is cross-sectional and correlational, these findings should be interpreted as associations rather than causal effects (see Limitations).

## Discussion

This study examined the structural relationships among psychological capital (PsyCap), psychological empowerment (PE), professional competence (PCo), and professional commitment among educational supervisors in Saudi Arabia. The findings are consistent with a dual-pathway model in which PsyCap is associated with professional commitment through two largely independent statistical pathways: a motivational pathway via psychological empowerment and a capability pathway via professional competence. By integrating these constructs within a single structural framework, this study extends existing literature, which has largely examined them in isolation, and offers a more comprehensive account of their associations with sustained professional engagement.

### Theoretical contribution: Integrating resource, motivation, and capability perspectives

One of the key contributions of this study lies in the integration of two complementary theoretical perspectives: Self-Determination Theory (SDT) and Conservation of Resources (COR) theory. SDT describes motivational processes associated with the satisfaction of autonomy, competence, and relatedness needs, whereas COR theory describes how psychological resources correspond with individuals’ capacity to maintain functioning under demanding conditions [8].

Rather than operating in isolation, these theories jointly explain the observed relationships in this study. PsyCap represents the core personal resource base (COR), psychological empowerment reflects motivational activation through need satisfaction (SDT), and professional competence reflects the accumulated capability that supports sustained professional functioning [7]. This integrated perspective addresses limitations in prior research, where these constructs have typically been examined separately, and provides a more coherent explanation of professional commitment in complex organisational contexts.

### Psychological capital as a foundational resource

Consistent with hypotheses H1 and H2, PsyCap showed strong positive associations with both psychological empowerment and professional competence. This finding reinforces the conceptualisation of PsyCap as a foundational psychological resource associated with both work-related perceptions and self-reported professional capability. In the context of educational supervision, PsyCap may represent an important internal asset during demanding and evolving roles. Supervisors with high self-efficacy may report greater confidence in evaluating instructional practices; those with strong hope may be more likely to set and pursue improvement goals; resilience may correspond with adaptation to policy changes and workload pressures; and optimism may accompany positive expectations during prolonged reform processes. PsyCap also showed a substantial total association with professional commitment. This pattern is consistent with emerging research indicating that psychological capital may buffer pathways through which workaholism and psychological distress are associated with burnout and cynicism [10].

### Psychological empowerment and professional commitment

The results showed that psychological empowerment had a strong direct association with professional commitment, supporting H4. This finding highlights the relevance of motivational processes to professional engagement. Supervisors who perceive their work as meaningful, experience autonomy in decision-making, and believe they have an impact on educational outcomes also tend to report stronger professional attachment [19,21]. From an SDT perspective, this pattern is consistent with the proposition that satisfaction of core psychological needs corresponds with intrinsic motivation and internalisation of professional roles. In practice, empowerment may coincide with movement beyond compliance-oriented behaviour toward more proactive engagement in teacher development and reform implementation [35,36]. However, empowerment was not significantly associated with competence, indicating that perceived motivation and self-reported capability may operate independently.

### The Non-Significant Path: Psychological Empowerment → Professional Competence

The non-significant relationship between psychological empowerment and professional competence represents one of the most theoretically important findings of this study. While prior research often assumes that empowered individuals are more likely to develop competence, the present results suggest that this relationship is not automatic and may be context-dependent [37].

Several explanations can be considered. First, the organisational structure of educational supervision in Saudi Arabia may limit opportunities for connecting perceptions of empowerment with skill development. Supervisors often operate within hierarchical systems with high administrative demands, where perceived autonomy may not correspond with access to professional-development resources or time for skill enhancement.

Second, a possible reverse temporal ordering should be considered: professional competence may precede stronger perceptions of empowerment rather than follow them. Supervisors who report higher professional competence may also feel more empowered because their expertise corresponds with greater confidence in decision-making and influence. This possibility cannot be tested with the cross-sectional data.

Third, the distinction between the competence-perception facet embedded within the empowerment measure and the separately self-reported professional competence scale may account for the absence of a significant relationship. Because both are self-reported rather than independently observed, this explanation remains speculative; supervisors may feel capable on the empowerment measure without this necessarily reflecting the same skills captured by the competence scale, particularly in resource-constrained environments. This finding contributes to the literature by challenging linear assumptions about empowerment and highlighting the need to consider structural and contextual conditions that enable or constrain competence development.

### Professional competence as a pathway to commitment

Professional competence was significantly and positively associated with professional commitment, supporting H5. This finding underscores the relevance of capability-based resources to professional engagement. Supervisors who report strong technical, interpersonal, and problem-solving skills may be better positioned to manage complex responsibilities and may also report greater confidence and professional identification. From a theoretical perspective, this association is consistent with self-efficacy and identity-based explanations: mastery experiences may correspond with confidence in professional abilities, while competence may coincide with a more coherent professional identity. The indirect association through competence further suggests that psychological resources and commitment are linked partly through supervisors’ reported skills and capabilities.

### A dual-pathway model of professional commitment

Taken together, the findings support a dual-pathway model in which PsyCap is associated with professional commitment through two distinct but complementary mechanisms: a motivational pathway (PsyCap → PE → commitment) and a capability pathway (PsyCap → PCo → commitment). Higher psychological resources were associated with greater meaning, autonomy, and perceived impact.

Psychological resources were also associated with professional mastery, which in turn corresponded with stronger commitment through competence and professional identity. The absence of a significant association between empowerment and competence indicates that these pathways operated largely independently [36]. This insight represents a key theoretical contribution because it suggests that motivation and capability may be treated as parallel processes rather than assumed to occur as sequential stages.

### Contextualising the Findings: Saudi Arabian Educational Reform

The findings must be interpreted within the broader context of Saudi Arabia’s educational transformation under Vision 2030. Supervisors operate within a system characterised by hierarchical governance structures, expanding responsibilities, and increasing accountability demands [4]. While reforms emphasise empowerment, participation, and professional development, implementation remains uneven. Supervisors may experience psychological empowerment in terms of perceived meaning and impact, yet still face constraints such as heavy workloads, limited training opportunities, and administrative demands that restrict competence development. Cultural factors may also play a role. In collectivist contexts, professional commitment may be influenced more by organisational alignment and social expectations than by individual career identity [38]. These contextual dynamics may help explain why empowerment is strongly associated with commitment but not with competence within this setting.

### Practical implications

The findings identify several candidate areas for future educational-policy and leadership research rather than demonstrating that particular interventions will be effective. First, psychological-capital-focused activities, including resilience training, coaching, goal-setting workshops, and leadership development, warrant testing in longitudinal or intervention studies. Second, empowerment-supportive practices—such as meaningful participation in decision-making, recognition of supervisors’ contributions, and appropriate professional discretion—may be evaluated as possible supports for professional commitment. Leadership approaches that combine humility, flexibility, and participation may also warrant examination in relation to readiness for change [39]. Third, professional-development investments, mentoring systems, and workload adjustments should be tested as possible supports for professional competence. Finally, structural constraints, including excessive supervisory loads and administrative demands, merit attention in future evaluations of how organisational conditions relate to motivation, competence, and commitment.

### Limitations and future research

Several limitations should be acknowledged when interpreting these findings.

First, the cross-sectional design precludes causal inference; all reported relationships are associations, and the direction and temporal ordering implied by the hypothesised model cannot be confirmed. Longitudinal or experimental designs are needed to establish temporal precedence among psychological capital, empowerment, competence, and commitment.

Second, all variables were measured via self-report, raising the possibility of common method bias, although Harman’s single-factor test and a comparison of single- versus multi-factor model fit suggested this was not a major concern. Future research should incorporate multi-source data, such as supervisor evaluations, peer or supervisee ratings, or objective performance indicators, to corroborate self-reported competence and commitment.

Third, three of the four constructs (psychological capital, psychological empowerment, and professional competence) were modelled using subscale-level composite indicators (parcels) rather than individual items, and professional commitment was represented as a single observed composite score rather than as a multi-indicator latent factor. This approach yielded well-fitting, parsimonious models and is a recognised practice for brief unidimensional scales, but it also means that item-level measurement error and possible multidimensionality within professional commitment could not be examined. Future research should test these relationships using full item-level measurement models where feasible.

Fourth, the sample was drawn using convenience sampling from a single administrative region (the Eastern Province of Saudi Arabia), albeit spanning 10 educational districts, which limits generalisability to supervisors in other regions or educational systems. Probability-based, multi-region sampling would strengthen external validity in future work.

Fifth, professional competence was assessed through supervisors’ own perceptions rather than objective performance indicators, and the non-significant association between psychological empowerment and competence may partly reflect this reliance on perceived rather than demonstrated competence, as well as potential conceptual overlap between empowerment and competence items. Future research should examine multidimensional operationalisations of empowerment and incorporate independent or objective measures of competence.

Sixth, demographic and professional-background variables (e.g., gender, years of experience, educational district) were described but not entered as covariates in the structural model; this choice reflects the study’s focus on testing the hypothesised psychological mechanisms rather than on estimating demographically adjusted effects, and future research could usefully test whether these associations vary across demographic subgroups.

Finally, future studies should investigate potential moderating variables, such as leadership style, organisational climate, and workload, and should extend the model to include downstream outcomes such as supervisory effectiveness and student achievement.

## Conclusion

This study examined the psychological mechanisms associated with professional commitment among educational supervisors by integrating psychological capital, psychological empowerment, and professional competence within a single structural model. The findings are consistent with psychological capital functioning as a foundational resource associated with professional commitment through both a direct pathway and two largely independent indirect pathways—one motivational (via empowerment) and one capability-based (via competence). By distinguishing these pathways, this study contributes to a more precise theoretical account of how psychological resources relate to professional commitment, and suggests that motivational perceptions alone may be insufficient for competence development in the absence of supportive organisational conditions. Because the design is cross-sectional and correlational, these conclusions should be treated as associational rather than causal, and as a basis for future longitudinal and intervention research rather than as direct evidence of intervention effectiveness. With that caveat, the findings may usefully inform future research on psychological-capital-focused interventions, empowerment-supportive leadership practices, and continuous professional-development systems as candidate strategies for supporting educational supervisors’ commitment, including within reform contexts such as Saudi Arabia’s Vision 2030.

## References

[1] Meyer JP, Allen NJ. A three-component conceptualization of organizational commitment. Hum Resour Manag Rev. 1991;1(1):61–89.

[2] Porter LW, Steers RM, Mowday RT, Boulian PV. Organizational commitment and turnover. J Appl Psychol. 1974;59(5):603–9.

[3] Amir M, Lipka O, Sarid M. Professional commitment and turnover intentions of elementary school teachers during educational crisis. Front Educ. 2025;10:1548359. doi: 10.3389/feduc.2025.1548359

[4] OECD. Education in Saudi Arabia. Paris: OECD Publishing. 2020. doi: 10.1787/76df15a2-en

[5] Kingdom of Saudi Arabia, Council of Economic and Development Affairs. Saudi Vision 2030. Riyadh: Government of Saudi Arabia. 2016. https://www.vision2030.gov.sa

[6] Aljaghthami EN. Psychological capital, leadership styles, and work engagement among women teachers in Saudi Arabia. Universiti Teknologi MARA. 2017.

[7] Deci EL, Ryan RM. Intrinsic motivation and self-determination in human behavior. New York: Springer. 1985.

[8] Hobfoll SE. Conservation of resources. A new attempt at conceptualizing stress. Am Psychol. 1989;44(3):513–24. doi: 10.1037//0003-066x.44.3.513 2648906

[9] Luthans F, Avolio BJ, Avey JB, Norman SM. Psychological capital: measurement and relationship with performance and satisfaction. Pers Psychol. 2007;60(3):541–72.

[10] Saeed I, Xigen W, Shah TA, Hussain I. Workaholism’s hidden cost: decoding burnout and cynicism through psychological distress and the buffering power of psychological capital. Psychol Health. 2026;:1–20. doi: 10.1080/08870446.2026.2620447 41586783

[11] Gong B, Chen X, Wang N, Zhan Y, Zhong H, Zhang R. Psychological capital, stress, micro-learning environment, and professional identity in nursing interns. Frontiers in Psychology. 2025;16:1458384. doi: 10.3389/fpsyg.2025.1458384 40166398 PMC11955965

[12] Avey JB, Reichard RJ, Luthans F, Mhatre KH. Meta-analysis of the impact of positive psychological capital on employee attitudes, behaviors, and performance. Hum Resour Dev Q. 2011;22(2):127–52. doi: 10.1002/hrdq.20070

[13] Abbas M, Raja U, Darr W, Bouckenooghe D. Combined effects of perceived politics and psychological capital on job satisfaction, turnover intentions, and performance. J Manage. 2014;40(7):1813–30. doi: 10.1177/0149206312455243

[14] Spreitzer GM. Psychological empowerment in the workplace: dimensions, measurement, and validation. Acad Manage J. 1995;38(5):1442–65.

[15] Zhang Y, Ishak ZB, Azam SBBM, Kang Y. A moderated mediation analysis of the relationship between psychological capital and professional commitment among teachers. Sci Rep. 2025;15(1):15021. doi: 10.1038/s41598-025-99497-5 40301492 PMC12041349

[16] Cervellione B, Lombardo EMC, Iacolino C. Psychological resources and interventions for teachers’ emotional competence and well-being: a systematic review. Front Psychol. 2025;16:1640968. doi: 10.3389/fpsyg.2025.1640968 40893859 PMC12394466

[17] Rodríguez-Aguirre C, De León DA, Garza-Olivares X. Academic burnout and psychological capital. In: IFE Conference Proceedings. 2025.

[18] Simons JC, Buitendach JH. Psychological capital, work engagement and organisational commitment amongst call centre employees in South Africa. SA j ind psychol. 2013;39(2). doi: 10.4102/sajip.v39i2.1071

[19] Bin Abdullah AGK, Almadhoun TZ, Ling YL. Organizational empowerment and commitment: the mediating effect of psychological empowerment. Asian J Soc Sci Arts Humanit. 2015;3(2):1–8.

[20] Puspaningtyas D. The effect of professional competence, work motivation, and work discipline on teacher performance: the mediation role of school culture. J Res Soc Sci Econ Manag. 2025;5(1):2923–39. doi: 10.59141/jrssem.v5i1.999

[21] Lin CP, Liu CM, Chan HT. Developing job performance: mediating effect of ethical climate and organisational identification. Pers Rev. 2022;51(2):750–69.

[22] Elsey V, Van der Heijden B, Smith MA, Moss M. Examining the role of employability as a mediator in the relationship between psychological capital and objective career success. Frontiers in Psychology. 2022;13:958226. doi: 10.3389/fpsyg.2022.958226 36591007 PMC9794865

[23] Al-Zahrani KA. Psychological capital as a predictor of electronic counseling tendency among student counselors. Mansoura Univ J Fac Educ. 2024;126(1):2–41. doi: 10.21608/maed.2024.377955

[24] Al Mahamid ASA. The relationship of psychological capital to emotional balance and professional motivation for educational counselors of Karak Governorate. Mu’tah University. 2022.

[25] Etikan I, Musa SA, Alkassim RS. Comparison of convenience sampling and purposive sampling. Am J Theor Appl Stat. 2016;5(1):1–4. doi: 10.11648/j.ajtas.20160501.11

[26] Kline RB. Principles and practice of structural equation modeling. 4th ed. New York: Guilford Press. 2016.

[27] Boomsma A, Hoogland JJ. The robustness of LISREL modeling revisited. In: Cudeck R, du Toit S, Sörbom D, editors. Structural equation models: present and future. Lincolnwood, IL: Scientific Software International. 2001. p. 139–68.

[28] Al-Yassin SMM. The effectiveness of a supervision program based on the discrimination model to improve perceived professional competence and quality of professional life. Mu’tah University. 2023.

[29] Almulla M. An investigation of teachers’ motivations for entering the teaching profession in Saudi Arabia. University of York. 2020.

[30] Satorra A, Bentler PM. Corrections to test statistics and standard errors in covariance structure analysis. In: von Eye A, Clogg CC, editors. Latent variable analysis. Thousand Oaks, CA: Sage. 1994. p. 399–419.

[31] Rhemtulla M, Brosseau-Liard PÉ, Savalei V. When can categorical variables be treated as continuous? A comparison of robust continuous and categorical SEM estimation methods under suboptimal conditions. Psychol Methods. 2012;17(3):354–73. doi: 10.1037/a0029315 22799625

[32] Fornell C, Larcker DF. Evaluating structural equation models with unobservable variables and measurement error. J Mark Res. 1981;18(1):39–50. doi: 10.2307/3151312

[33] Hair JF, Black WC, Babin BJ, Anderson RE. Multivariate data analysis. 7th ed. Upper Saddle River, NJ: Pearson. 2010.

[34] Podsakoff PM, MacKenzie SB, Lee J-Y, Podsakoff NP. Common method biases in behavioral research: a critical review of the literature and recommended remedies. J Appl Psychol. 2003;88(5):879–903. doi: 10.1037/0021-9010.88.5.879 14516251

[35] Barattucci M, Teresi M, Di Berardino C, Di Crescenzo A, Pagliaro S. The relationship between psychological empowerment and organisational identification: a network analysis approach. Int J Appl Posit Psychol. 2025;10(2):35. doi: 10.1007/s41042-025-00229-x

[36] Creon LE, Schermuly CC. Feeling safe to be empowered: psychological safety and psychological empowerment in threatening work environments. Ger J Hum Resour Manag. 2025;39(2):148–76. doi: 10.1177/21582440241284536

[37] Oliveira M, Andrade JR, Ratten V, Santos E. Psychological empowerment for the future of work. Glob Bus Organ Excell. 2023;42(5):65–78.

[38] Ahmed EI. Teacher empowerment and organisational citizenship behaviour in public schools in Saudi Arabia. Manag Educ. 2024;38(1):22–9. doi: 10.1177/08920206211030929

[39] Saeed I, Wang X, Azizi N, Shah TA, Raza MI. Leading with humility: how leadership humility drives change-oriented organizational citizenship behavior through readiness to change, with flexible work practices as a moderator. Psychological Reports. 2025. doi: 10.1177/00332941251399154 41255087

